# Supplementary material for: A multiphase theory for spreading microbial swarms and films
Source: eLife. 2019 Apr 30;8:e42697. doi: 10.7554/eLife.42697 (PMC6491038; doi:10.7554/eLife.42697)
Supplement: Transparent reporting form [file elife-42697-transrepform.docx]

***eLife’s* transparent reporting form**

We encourage authors to provide detailed information *within their submission* to facilitate the interpretation and replication of experiments. Authors can upload supporting documentation to indicate the use of appropriate reporting guidelines for health-related research (see [EQUATOR Network](http://www.equator-network.org/%20)), life science research (see the [BioSharing Information Resource](https://biosharing.org/)), or the [ARRIVE guidelines](http://www.plosbiology.org/article/info:doi/10.1371/journal.pbio.1000412) for reporting work involving animal research. Where applicable, authors should refer to any relevant reporting standards documents in this form.

If you have any questions, please consult our Journal Policies and/or contact us: [editorial@elifesciences.org](mailto:editorial@elifesciences.org).

**Sample-size estimation**

- You should state whether an appropriate sample size was computed when the study was being designed
- You should state the statistical method of sample size computation and any required assumptions
- If no explicit power analysis was used, you should describe how you decided what sample (replicate) size (number) to use

1) For the illustrative example in Fig. 1, we use a single sample grown under the conditions described in the Materials and Methods section in the Appendix.

2) To determine the steady state *B. subtilis* swarm expansion velocity in Figure 3, three replicates were used to determine the deviation in steady state speed. To fit the biofilm velocity speeds, we use previously published experimental data from Srinivasan et al, Biophysical Journal, 2018, as cited in Figure 4.

3) The simulations are fully deterministic, therefore a single simulation with a given set of parameters produces the same reproducible results.

Please outline where this information can be found within the submission (e.g., sections or figure legends), or explain why this information doesn’t apply to your submission:

**Replicates**

- You should report how often each experiment was performed
- You should include a definition of biological versus technical replication
- The data obtained should be provided and sufficient information should be provided to indicate the number of independent biological and/or technical replicates
- If you encountered any outliers, you should describe how these were handled
- Criteria for exclusion/inclusion of data should be clearly stated
- High-throughput sequence data should be uploaded before submission, with a private link for reviewers provided (these are available from both GEO and ArrayExpress)

1) For the swarming experiments on *B. subtilis*, swarm expansion speeds were measured on 3 agarose swarm plates from the same starter culture.

2) The experimental data for the osmotic pressure in *E. coli* swarms is reproduced from the previously published work of Ping et al, Biophysical Journal, 2014, as cited in Figure 3, and is quantitatively compared to our numerical simulations.

3) The values of the velocity profiles and the EPS production profiles in *B. subtilis* biofilms are reproduced from the previously published work of Srinivasan et al, Biophysical Journal, 2018 as cited in Figure 4, and are quantitatively compared to our numerical simulations.

Please outline where this information can be found within the submission (e.g., sections or figure legends), or explain why this information doesn’t apply to your submission:

**Statistical reporting**

- Statistical analysis methods should be described and justified
- Raw data should be presented in figures whenever informative to do so (typically when N per group is less than 10)
- For each experiment, you should identify the statistical tests used, exact values of N, definitions of center, methods of multiple test correction, and dispersion and precision measures (e.g., mean, median, SD, SEM, confidence intervals; and, for the major substantive results, a measure of effect size (e.g., Pearson's r, Cohen's d)
- Report exact p-values wherever possible alongside the summary statistics and 95% confidence intervals. These should be reported for all key questions and not only when the p-value is less than 0.05.

Please outline where this information can be found within the submission (e.g., sections or figure legends), or explain why this information doesn’t apply to your submission:

For a single representative *B. subtilis* swarm experiment, the expansion velocity of the swarm was measured at intervals of 10 s over a 10 min period as described in the inset of Figure 1. The mean velocity over these technical replicates was measured as V=2 mm/h. A similar technique was applied to three biological replicates to determine the standard deviation as V=0.5 mm/h

For the biofilm velocity profiles, experimental data is reproduced from Srinivasan et al., 2018 and indicates median expansion velocities (filled circles), the 25th to 75th percentile velocities (filled box), and extreme values (vertical lines) as indicated in the caption of Fig 4

(For large datasets, or papers with a very large number of statistical tests, you may upload a single table file with tests, Ns, etc., with reference to sections in the manuscript.)

**Group allocation**

- Indicate how samples were allocated into experimental groups (in the case of clinical studies, please specify allocation to treatment method); if randomization was used, please also state if restricted randomization was applied
- Indicate if masking was used during group allocation, data collection and/or data analysis

Please outline where this information can be found within the submission (e.g., sections or figure legends), or explain why this information doesn’t apply to your submission:

N/A – bacteria do not need care.

**Additional data files (“source data”)**

- We encourage you to upload relevant additional data files, such as numerical data that are represented as a graph in a figure, or as a summary table
- Where provided, these should be in the most useful format, and they can be uploaded as “Source data” files linked to a main figure or table
- Include model definition files including the full list of parameters used
- Include code used for data analysis (e.g., R, MatLab)
- Avoid stating that data files are “available upon request”

The COMSOL files used for numerical simulations whose results are plotted in Figure 3 and Figure 4 are provided as additional data files.

The full list of parameters and numerical values used in the simulations is provided as a Table in the Appendix.

Please indicate the figures or tables for which source data files have been provided:
